# Robust succinic acid production from crude glycerol using engineered *Yarrowia lipolytica*

**DOI:** 10.1186/s13068-016-0597-8

**Published:** 2016-08-30

**Authors:** Cuijuan Gao, Xiaofeng Yang, Huaimin Wang, Cristina Perez Rivero, Chong Li, Zhiyong Cui, Qingsheng Qi, Carol Sze Ki Lin

**Affiliations:** 1 School of Energy and Environment, City University of Hong Kong, Tat Chee Avenue, Kowloon, Hong Kong; 2 State Key Laboratory of Microbial Technology, Shandong University, Jinan, 250100 People’s Republic of China; 3 School of Life Science, Linyi University, Linyi, 276005 People’s Republic of China; 4 School of Bioscience and Bioengineering, South China University of Technology, Guangzhou, 510006 People’s Republic of China; 5 School of Chemical Engineering and Analytical Science, The University of Manchester, Manchester, UK

**Keywords:** *Yarrowia lipolytica*, Metabolic engineering, Succinic acid, Crude glycerol, Fed-batch fermentation

## Abstract

**Background:**

Integrating waste management with fuels and chemical production is considered to address the food waste problem and oil crisis. Approximately, 600 million tonnes crude glycerol is produced from the biodiesel industry annually, which is a top renewable feedstock for succinic acid production. To meet the increasing demand for succinic acid production, the development of more efficient and cost-effective production methods is urgently needed. Herein, we have proposed a new strategy for integration of both biodiesel and SA production in a biorefinery unit by construction of an aerobic yeast *Yarrowia lipolytica* with a deletion in the gene coding succinate dehydrogenase subunit 5.

**Results:**

Robust succinic acid production by an engineered yeast *Y. lipolytica* from crude glycerol without pre-treatment was demonstrated. Diversion of metabolic flow from tricarboxylic acid cycle led to the success in generating a succinic acid producer *Y. lipolytica* PGC01003. The fermentation media and conditions were optimized, which resulted in 43 g L^−1^ succinic acid production from crude glycerol. Using the fed-batch strategy in 2.5 L fermenter, up to 160 g L^−1^ SA was yielded, indicating the great industrial potential.

**Conclusions:**

Inactivation of SDH5 in *Y. lipolytica* Po1f led to succinic acid accumulation and secretion significantly. To our best knowledge, this is the highest titer obtained in fermentation on succinic acid production. In addition, the performance of batch and fed-batch fermentation showed high tolerance and yield on biodiesel by-product crude glycerol. All these results indicated that PGC01003 is a promising microbial factorial cell for the highly efficient strategy solving the environmental problem in connection with the production of value-added product.

**Electronic supplementary material:**

The online version of this article (doi:10.1186/s13068-016-0597-8) contains supplementary material, which is available to authorized users.

## Background

Since fossil fuel resource is in the trend of depletion, it becomes apparent that a switch from petrochemical-based chemical production industry toward a bio-based and carbon neutral process is inevitable. Robust and efficient microbes are urgently needed for the cost-effective biosynthesis of valuable chemicals. *Yarrowia lipolytica* is a strictly aerobic microorganism and one of the most extensively studied “nonconventional” yeasts, which justifies efforts for its use in industry as a robust producer as well as in molecular biology and genetics studies [1]. It can utilise a large variety of substrates, including glucose, glycerol, ethanol, acetate and also hydrophobic substance such as lipids and fatty acids [2]. Besides, it exhibits excellent tolerance to environmental stress in the existence of salt, low temperatures, acidic and alkaline pH. Furthermore, *Y. lipolytica* presents the ability to produce and secrete a great variety of organic acids, including TCA cycle intermediates, like citric acid, isocitric acid, α-ketoglutaric acid and succinic acid (SA) [3–6].

SA is one of the most important building block chemicals and was identified as one of the top twelve potential chemical building blocks for the future by the US Department of Energy [7]. Due to its versatile application, the global SA market grows rapidly, and the market value is expected to reach US$ 0.54 billion in 2020 by Weastra [8]. Currently, SA is commonly refined from petroleum, but is a natural intermediate in the metabolic pathways of many microorganisms. Additionally, the total addressable market volume for bio-based SA is estimated to reach US$ 14.1 billion. Therefore, petroleum-based chemical production should be shifted to biotechnological processes for a long-term consideration of environment.

For industrial scale bio-based SA production, raw materials account for the major operational cost. Establishment of a bio-based and green economy depends on the availability of inexpensive organic carbon compounds. Crude glycerol is one of the low-cost waste materials, which is formed from the production of biodiesel. Due to the raise of biodiesel production, glycerol became a highly available substrate for bio-based chemicals production. For each 10 L of biodiesel produced, almost 1 L glycerol is produced as by-product, turning into a burden for the industry [9]. Around 600 million tonnes crude glycerol is produced annually [10, 11]. In terms of commercial feasibility, glycerol is ranked as the second top feedstock in bio-SA production among 15 types of renewable biomass [12].

Using crude glycerol as substrate for SA production, it is not only helpful to utilise the waste stream in biodiesel production but also beneficial to the environment. The pathways relating SA biosynthesis from glycerol are shown in Additional file 1: Figure S1. There is no massive pre-disposition for SA accumulation naturally in wild type of *Y. lipolytica* as SA is an intermediate of TCA cycle. Therefore, strategies should be considered to block its metabolism.

In this study, the gene encoding a subunit of succinate dehydrogenase complex (SDH5) that is a highly conserved mitochondrial protein required for SDH-dependent respiration and for flavination (incorporation of the flavin adenine dinucleotide cofactor) was knocked out in *Y. lipolytica*. SA production using the engineered strain was investigated with respect to its tolerance and productivity in crude glycerol, which was obtained directly from ASB Biodiesel Plant in Hong Kong [13]. The proposed bioprocess could be integrated with a traditional transesterification process for the production of biodiesel and SA.

## Results and discussion

### Engineering *Y. lipolytica* for SA production by deletion of *Ylsdh5*

*Yarrowia lipolytica* owns the capability to produce organic acid, such as citric acid, isocitric acid and 2-oxoglutaric acid [14, 15]. Production of succinic acid using *Y. lipolytica* was adopted previously by combination of microbial synthesis of α-ketoglutaric acid and subsequent chemically assisted decarboxylation of α-ketoglutaric acid by hydrogen peroxide to SA [16].

**Fig. 1:**
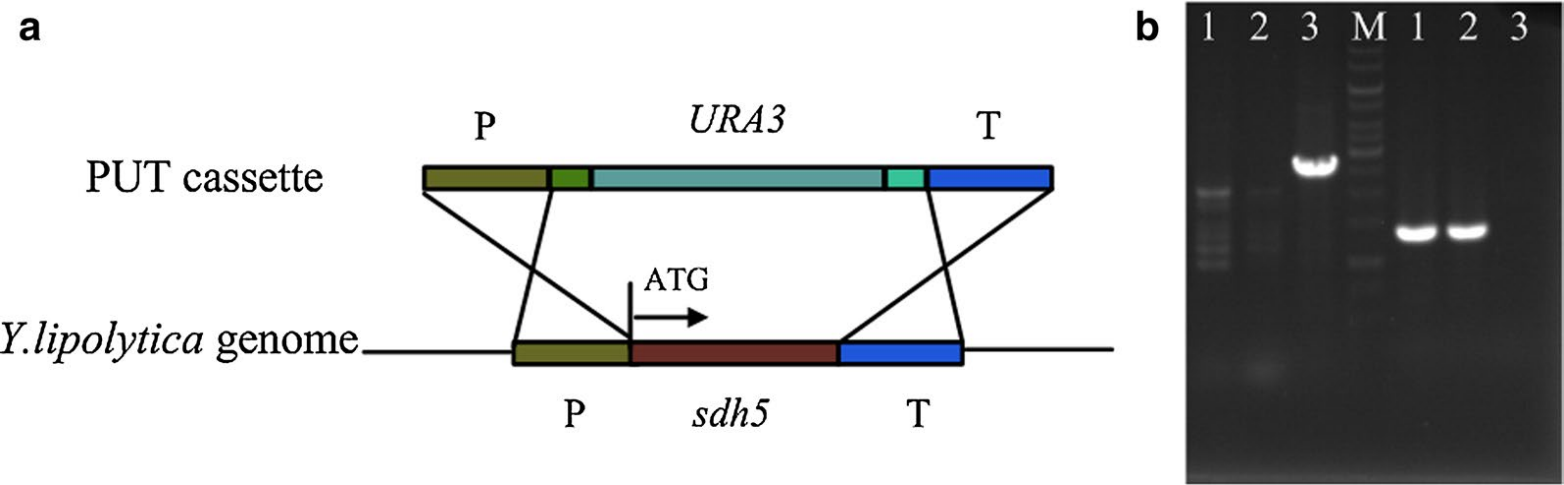
Schematic view of PCR-based gene disruption (**a**) and the mutant confirmation by PCR (**b**). **a** The PUT cassette obtained via PCR using Up-F/Down-R was used to generate the *sdh5*::*URA3* disruption by homologous recombination. Clones that contain URA_3_ can grow on the selected plate. **b** Diagnostic PCR using extracted genomic DNA of the transformants as template with two sets of primer pairs of chrom-F/ura-R (*left*) and chrom-F/chrom-R (*right*). *M* 1 kb DNA marker ladder (MBI). *1*, *2*, *3* were three independent transformants. Transformant 3 was verified to be positive

In cell, as an intermediate of TCA cycle, SA is formed from α-ketoglutaric acid through α-ketoglutarate dehydrogenase and from isocitric acid through isocitrate lyase, and subsequently depleted by the catalysis of succinate dehydrogenase complex (SDH). SDH, also known as complex II or succinate-ubiquinone oxidoreductase, participates in both the electron transport chain and tricarboxylic acid cycle which oxidizes SA to fumaric acid with the reduction of the mobile electron carrier ubiquinone to ubiquinol [17]. The SDH complex consists of five subunits, of which the subunits SDH1 and SDH2 formed the catalytic dimer, anchored by the subunits SDH3 and SDH4 in the mitochondrial membrane [18, 19]. The fifth subunit SDH5, a highly conserved mitochondrial protein in SDH complex, is required for SDH activity and stability [18]. Recently, reduction or loss of SDH enzyme activity in *Y. lipolytica* was explored through impairing *sdh1*/*sdh2* gene or exchanging native promoter of *sdh2* gene with a weak promoter [5, 20]. These recombinant strains can accumulate about 4–5 g L^−1^ SA using glycerol as substrate in unbaffled flasks. Herein, the activity of SDH was interfered by deletion of *Ylsdh5* gene. The PUT cassette for homologous substitution onto SDH5 locus *Ylsdh5* (YALI0F11957 g) of *Y. lipolytica* Po1f genome was obtained by PCR amplification and was transformed into the competent cell (Fig. 1a). The positive clone was obtained after 3 days cultivation and was verified by diagnostic PCR (Fig. 1b) and sequencing. The *Ylsdh5* deleted strain and the parent strain Po1f were then cultivated in YPG with glycerol as carbon source for SDH activity determination. Po1f showed an average specific SDH activity of 146 ± 2 U, whereas the *Ylsdh5* deleted strain lacked SDH activity (0.9 ± 1.4 U). The resulted mutant, designated as PGC01003 was used for the evaluation of SA production.

### Evaluation of SA production in *Y. lipolytica* PGC01003

**Fig. 2:**
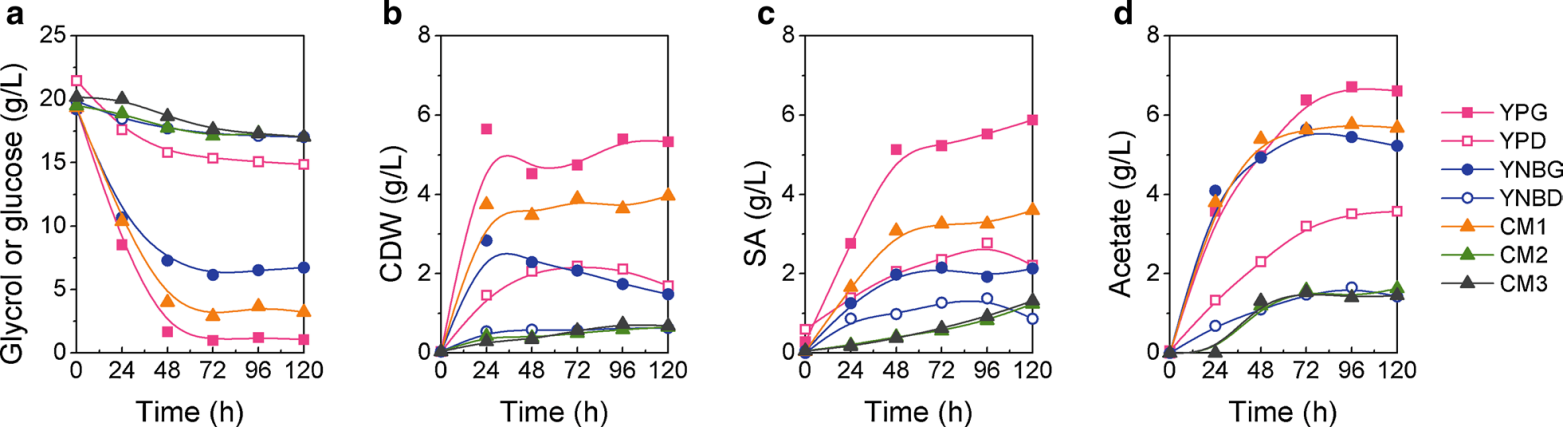
Fermentation profiles of PGC01003 in different media. The data were calculated from two independent experiments. **a** Residual glycerol or glucose, **b** CDW, **c** succinic acid, **d** acetic acid

The PGC01003 strain was evaluated with regards to its growth and substrate consumption in seven media comprising YPG, YPD, YNBG, YNBD, CM1, CM2 and CM3 in shaking flasks. As shown in Fig. 2, YPG was the optimum medium for cell growth and SA production (Fig. 2b, c). The carbon source consumption rate in YPG medium was the fastest among all the media (Fig. 2a), whereas yeast extract and tryptone are beneficial for the growth of PGC01003 (Fig. 2). However, only less than 6.6 g L^−1^ and 2.8 g L^−1^ glucose were consumed in YPD and YNBD after 120 h cultivation, respectively (Fig. 2a), indicated PGC01003 had low cell activity in glucose-based medium. Nevertheless, the PGC01003 strain secreted much acetic acid under various cultivation media (Fig. 2d). Therefore, the PGC01003 strain was compared with the control strain Po1g in YPG medium containing 2 % (w/v) glycerol. As shown in Table 1, PGC01003 grew slower and showed less biomass than Po1g, in which the maximum specific growth rate (*µ*_max_) was 0.40 and 0.53 h^−1^, respectively. Both strains consumed all glycerol completely after 72 h. Meanwhile, PGC01003 produced 5.5 g L^−1^ SA, which was 13 times more than that of Po1g.

**Fig. 3:**
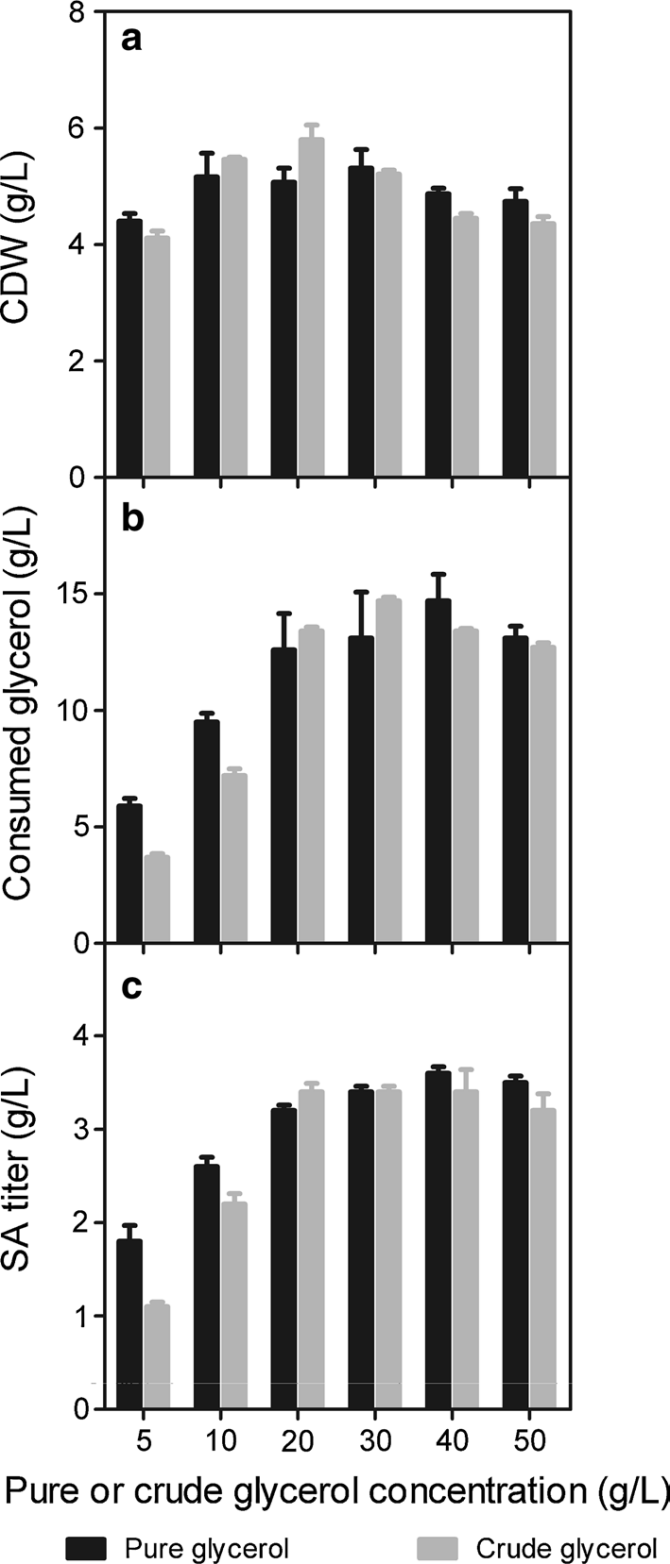
Comparison of CDW (**a**), consumed glycerol (**b**) and SA titer (**c**) by *Y. lipolytica* PGC01003 strain in various concentrations of crude glycerol and pure glycerol. Fermentation was carried out in shaking flask for 48 h

**Table 1:** Comparison of *Y. lipolytica* Po1g and PGC01003 strains in shaking flask

	Fermentation time (h)	Glycerol consumption rate (g L^−1^ h^−1^)	*μ*_max_ (h^−1^)^a^	CDW (g L^−1^)	C_AC_ (g L^−1^)^b^	C_SA_ (g L^−1^)^c^	SA productivity (g L^−1^ h^−1^)	Y_SA_ (g g^−1^)^d^
Po1 g	72	0.84	0.53	13.9	0.02	0.44	_	0.02
PGC01003	72	1.12	0.40	5.6	5.70	5.51	0.08	0.24

^a^ *μ*_max_ is the maximum specific growth rate

^b^C_AC_ is the concentration of acetic acid in the fermentation broth

^c^C_SA_ is the concentration of succinic acid in the fermentation broth

^d^Y_SA_ is the yield of succinic acid per gram of consumed glycerol

The obvious SA accumulation indicated the pathway from SA to fumaric acid was blocked by deletion of *Ylsdh5*. Compare to the loss of ability to grow in glucose after *sdh1* and/or *sdh2* deletion [20], the *sdh5* deleted strain PGC01003 demonstrated a weak growth in glucose (Fig. 3). Apart from SA, the PGC01003 strain also accumulated 5.7 g L^−1^ acetic acid, which was not found in the control strain. Jost et al. [5] also reported the *sdh2* deleted strain secreted acetic acid. This acetic acid overflow was expected due to the metabolic flux between glycolysis and TCA cycle became imbalance.

### Evaluation of crude glycerol as carbon source for SA production

#### Comparison of the performance of PGC01003 in the presence of pure and crude glycerol

We subsequently evaluated the potential utilization of crude glycerol for SA production by comparing the performance of PGC01003. PGC01003 strain showed similar growth characteristics in both crude and pure glycerol from 5 to 50 g L^−1^ initial concentrations (Fig. 3). The final CDW have no significant difference between crude and pure glycerol after 48 h cultivation (Fig. 3a). With the increasing amount of initial glycerol concentration, the consumed glycerol first increased and then decreased slightly in both media (Fig. 3b). The maximum SA titer of 3.6 and 3.4 g L^−1^ SA was obtained in 40 g L^−1^ pure glycerol and 30 g L^−1^ crude glycerol, respectively (Fig. 3b, c). We did not observe any inhibition phenomena when crude glycerol was used as carbon source, indicating *Y. lipolytica* has high tolerance to the residual inhibitors in the biodiesel production process. Additionally, the methanol inhibition experiment indicated that the methanol content in the crude glycerol from the ASB Biodiesel Plant (<2 g/L) does not inhibit the growth of *Y. lipolytica*. Interestingly, the SA production was slightly enhanced when the supplemented methanol increased from 0 to 20 g/L (data not shown).

#### Effect of pH and aeration on SA production in fermenter

**Fig. 4:**
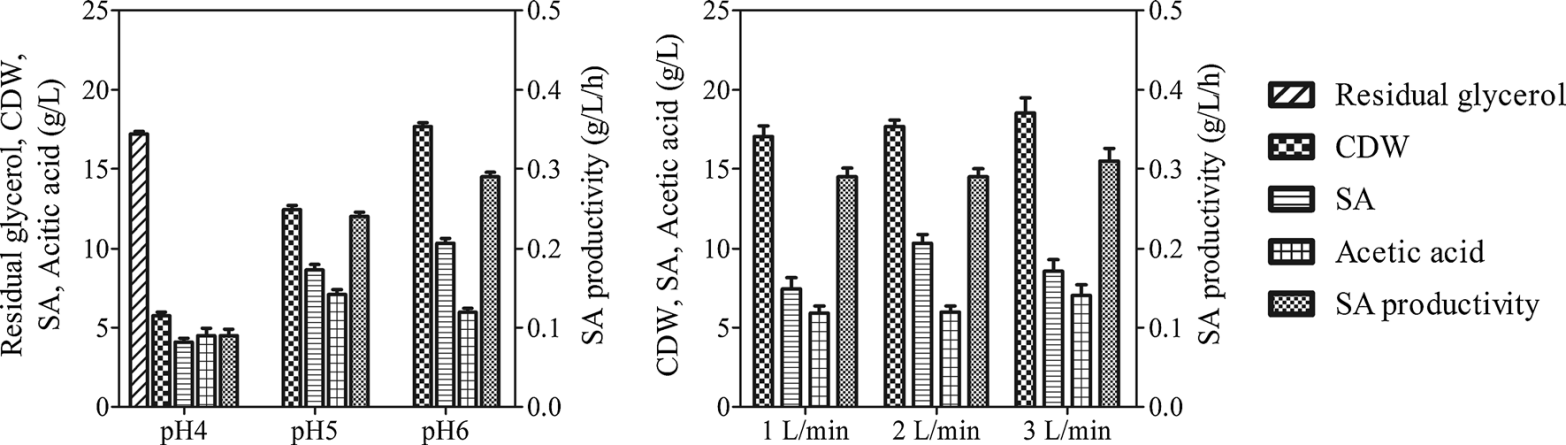
Comparison of cell growth and SA production by *Y. lipolytica* PGC01003 in batch fermentation at **a** various pH and **b** various aeration rate

To investigate pH and aeration effects on SA production, fermentations were conducted in 2.5-L benchtop fermenter with controlled system. As shown in Fig. 4a, the glycerol was completely depleted at pH 5.0 and 6.0 after 30 h cultivation. The highest biomass of 17.7 g L^−1^ was obtained at pH 6, which also achieved the highest SA production of 10.3 g L^−1^ SA with a productivity of 0.29 g L^−1^ h^−1^. However, the acetic acid secretion of 6.0 g L^−1^ at pH 6 was also less than the amount at pH 5 (7.1 g L^−1^). *Y. lipolytica* PGC01003 could also produce SA at low pH of 4.0. After 48 h cultivation, half of glycerol has been consumed to generate 5.8 g L^−1^ biomass and 4.1 g L^−1^ SA.

The effect of aeration on SA production was then studied in 2.5-L fermenter with aeration rate at 1.0, 2.0 and 3.0 L min^−1^, respectively (Fig. 4b). The dissolved oxygen decreased rapidly when cultures entered into the exponential growth phase. The oxygen limitation occurred at 1.0 and 2.0 L min^−1^ aeration. The highest CDW of 18.5 g L^−1^ was obtained in 3.0 L min^−1^ aeration. Meanwhile, the 2.0 L min^−1^ of aeration achieved the maximum titer of 10.3 g L^−1^ SA with low acetic acid secretion. Under aerobic conditions, NAD^+^ is regenerated from NADH by the reduction of oxygen, and the kinetic of redox is associated with the extracellular dissolved oxygen [21]. Therefore, the inappropriate dissolved oxygen level would result in an imbalance between the carbon source uptake and its conversion into biomass and SA, and then bypassing pyruvate from the TCA cycle to acetic acid [22]. Results from this study indicated that aeration rate of 2 L min^−1^ was a favourable condition for SA production.

### Optimization of the initial crude glycerol concentration in fermenter

**Fig. 5:**
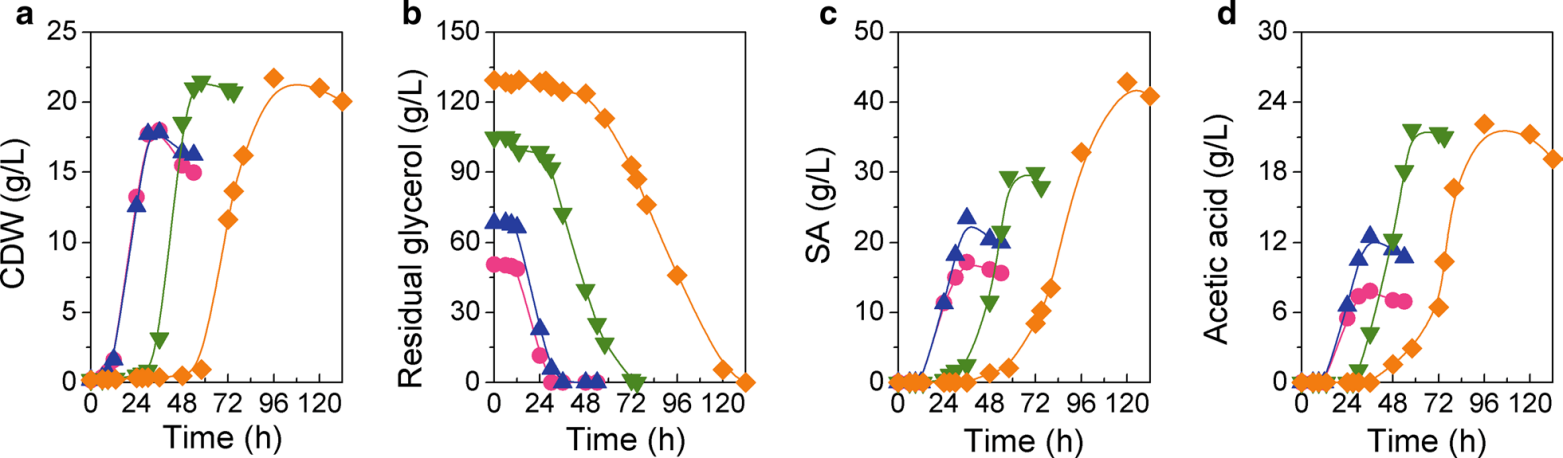
Kinetic profiles of cell growth and metabolites formation of *Y. lipolytica* PGC01003 strain at different initial crude glycerol of 75 g/L (*pink*, *circle*), 100 g/L (*blue*, *upper triangle*), 150 g/L (*olive*, *lower triangle*) and 200 g/L (*orange*, *diamond*)

*Yarrowia lipolytica* has been demonstrated have high tolerance of 150 g L^−1^ of initial crude glycerol in citric acid production [23, 24]. Although SA production by engineered *Y. lipolytica* was reported before, no more than 50 g L^−1^ initial glycerol concentration was used [5, 20]. The initial concentration of crude glycerol was optimized from 75 to 200 g L^−1^ in batch fermentations to improve SA fermentation performance. Figure 5 showed the fermentation kinetic profiles in various initial glycerol concentrations. Although the lag time elongated in 150 and 200 g L^−1^ crude glycerol, all glycerol was completely consumed by the PGC01003, and CDW, SA titer and acetic acid titer were positively correlated with the initial glycerol concentration. The highest SA production of 42.9 g L^−1^ was obtained from 200 g L^−1^ initial crude glycerol (equivalent to 129.4 g L^−1^ of pure glycerol) with the yield of 0.33 g/g glycerol, which represents 51.7 % of theoretical yield [20]. Fermentation with initial crude glycerol of 100 g L^−1^ led to the highest *μ*_max_, glycerol consumption rate, SA productivity and yield of 0.28 h^−1^,1.9 g^−1^ h^−1^, 0.65 g L^−1^ h^−1^, and 0.34 g g^−1^ glycerol (53.0 % of theoretical yield), respectively.

Glycerol conversion was always impaired by the redox imbalance during biomass formation [25], led to controlled glycerol at low concentration in SA production usually. Moreover, crude glycerol concentration was controlled under 5 g L^−1^ by continuous cultivation approach when using *Basfia succiniciproducens* DD1 [26]. Jost et al. [5] pointed out that glycerol concentration should not exceed 40 g L^−1^ for an engineered *Y. lipolytica* in SA fermentation. The experiment indicated that *Y. lipolytica* PGC01003 is able to grow well under high glycerol concentration, which would increase the SA productivity and facilitate the production process.

### Highly efficient SA production using fed-batch fermentation strategy

**Fig. 6:**
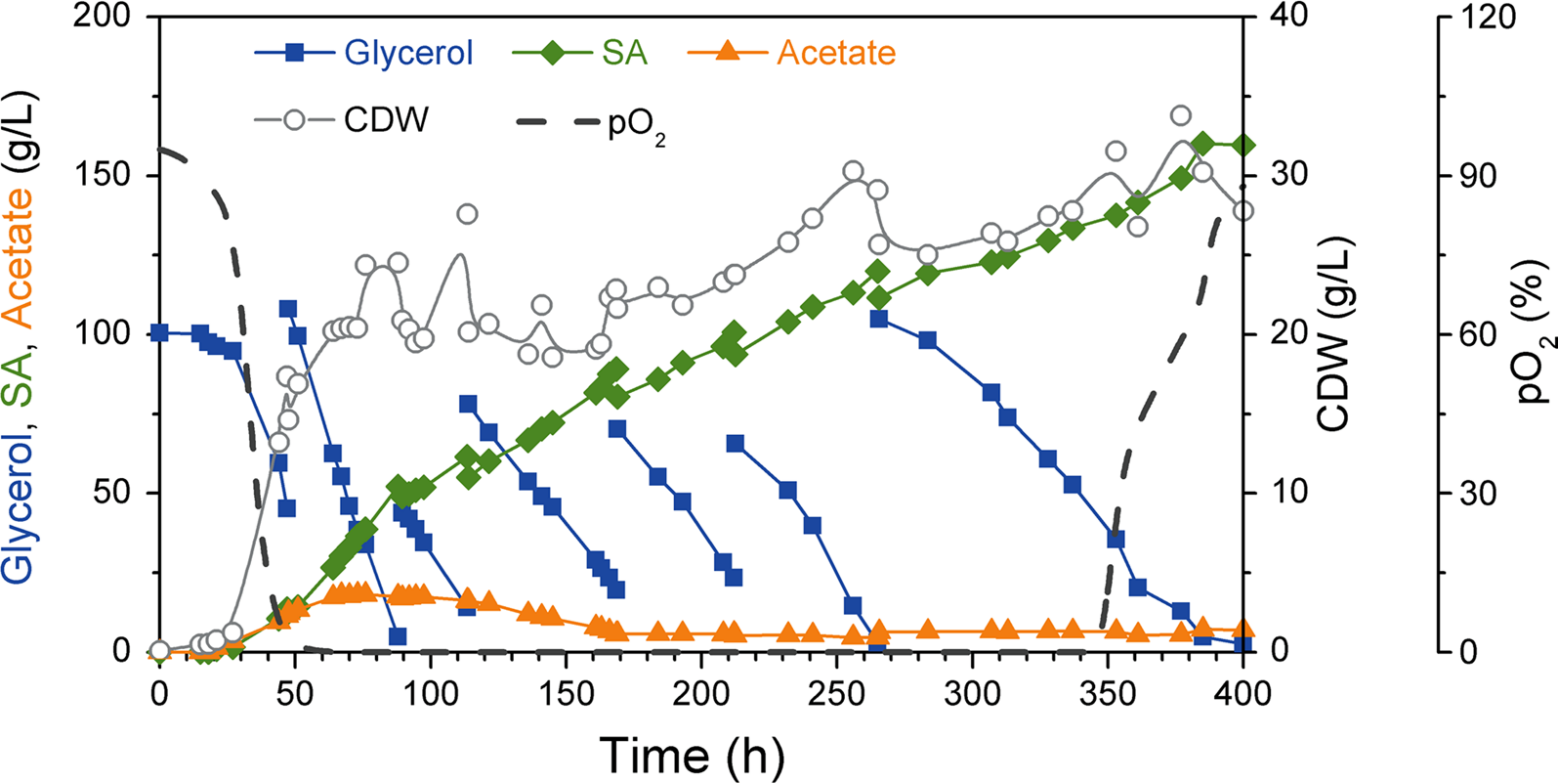
Fed-batch fermentation profile of SA production

To achieve high SA yield, fed-batch fermentation was carried out. The initial glycerol concentration was set at 100 g L^−1^ and 100–150 mL crude glycerol was fed from 750 g L^−1^ stock when the glycerol concentration dropped below 15 g L^−1^. The dissolved oxygen was completely depleted from 48 to 348 h, indicated that the cells have high oxygen uptake rate. Although the oxygen was limited, the biomass still slowly increased to 33.8 g L^−1^ with high glycerol consumption rate and SA productivity, which indicated the cell activity was maintained at high level during the whole process (Fig. 6). After 400 h cultivation with six times feeding, the final SA production was up to 160.2 g L^−1^. To our knowledge, this is the highest fermentative SA production achieved so far (Table 2). Moreover, the average SA productivity was up to 0.40 g L^−1^ h^−1^, which is significantly higher as compare to the previous yeast fermentation using *Saccharomyces cerevisiae* or *Y. lipolytica*, which were 0.12 g L^−1^ h^−1^ [27] and 0.27 g L^−1^ h^−1^ [20], respectively. The final SA yield was 0.40 g g^−1^ glycerol, representing 62.4 % of theoretical yield, which also have a significant increase compared to the highest yield in *S. cerevisiae* [27]. This result confirmed that the genetically modified *Y. lipolytica* PGC01003 strain can tolerate very high SA concentration and has great potential in fermentative SA production.

**Table 2:** Comparison of SA production by fed-batch fermentation strategy

Microorganism	Carbon source	SA titer (g L^−1^)	SA yield (g g^−1^)	SA productivity (g L^−1^ h^−1^)	References
*A. succinogenes* GXAS137	Sugarcane molasses	64.7	0.81	1.3	[32]
*C. glutamicum* CGMCC1006-Δ*ldhA*	Sugarcane molasses	35.1	0.37	0.65	[34]
*E. coli* BA305	Sugarcane bagasse	39.3	N/A	0.33	[35]
*E. coli* KJ122	Cassava pulp	98.6	0.72	1.03	[36]
*A. succinogenes* ATCC 55618	Rapeseed meal	23.4	N/A	0.33	[37]
*A. succinogenes* CGMCC1593	Wheat straw	53.2	N/A	1.21	[38]
*A. succiniciproducens*	Whey	34.7	0.91	1.02	[39]
*C. glutamicum* Δ*ldhA*-pCRA717	Glucose	146	0.92	3.17	[41]
*A. succinogenes* ATCC 55618	Glycerol	49.6	0.87	2.31	[25]
*E. coli* E2-Δsdh-ppc-sucAB	Glycerol	43.2	N/A	0.77	[40]
*Y. lipolytica* H222-AZ2	Glycerol	25	0.26	0.15	[5]
*Y. lipolytica* PGC01003	Crude glycerol	160.2	0.4	0.4	This study

After the second feeding, the glycerol consumption rate kept stable at 1.1 ± 0.3 g L^−1^ h^−1^, while the SA productivity dropped to 0.33 ± 0.09 g L^−1^ h^−1^ and then increased to 1.1 ± 0.2 g L^−1^ h^−1^ until next feeding (Additional file 2: Figure S2). The SA yield was slightly increased from 0.23 to 0.40 g g^−1^ glycerol during the fed-batch fermentation (Additional file 2: Figure S2C). Moreover, the acetic acid concentration increased at the beginning and stabilized at 17–18 g L^−1^ during 64–98 h, and finally the acetic acid dropped to 5–7 g L^−1^ after 166 h. It showed that acetic acid could be utilised as a carbon source in *Y. lipolytica* PGC01003 (Fig. 6) which was also observed in previous studies [28, 29]. Fermentation ended at 400 h and 1.2 L of fermentation broth was collected. A total of 105 g SA was recovered with 98.2 % purity and 54.6 % SA recovery yield by the vacuum distillation-crystallisation method [30].

Fed-batch fermentation has been extensively studied in many SA producer *Actinobacillus succinogenes* and *A. succiniciproducens*, or genetic modified strains, such as *Escherichia coli*, *Corynebacterium glutamicum* and *Y. lipolytica*, which has been demonstrated to be a useful strategy for enhancing the SA production. As shown in Table 2, several studies focused on fed-batch fermentation from renewable feedstock derived from agricultural residues, food supply chain or biodiesel production in SA production. Sugarcane molasses have been fermented into SA by *A. succinogenes* [31–33] and *C. glutamicum* [34] via fed-batch fermentation. By supplementing a mixture of corn steep liquor powder and peanut meal as nitrogen sources, up to 64.7 g L^−1^ SA was obtained with the yield of 0.81 g g^−1^ sugarcane molasses by *A. succinogenes* [32]. Other feedstocks, such as sugarcane bagasse [35], cassava pulp [36], rapeseed meal [37], wheat straw [38], whey [39] and glycerol [5, 25, 40] have been used also for the SA production via fed-batch fermentation.

One of the most crucial issues in platform chemicals production is to achieve high product titer [7]. High SA titer has been reported via fed-batch fermentation of *C. glutamicum* ΔldhA-pCRA717 mutant, which resulted 146 g L^−1^ of SA from pure glucose via two-stage fermentation process [41]. In this study, 160.2 g L^−1^ SA was obtained from crude glycerol by a genetic modified *Y. lipolytica* PGC01003 strain, which is the highest SA titer achieved so far. *Y. lipolytica* would be a promising industrial host for SA production from renewable feedstock. Results from this study successfully demonstrated that the co-production of biodiesel and a platform chemical would be a technically feasible approach, in which the valorisation of crude glycerol as a by-product from transesterification enables the sustainable production of SA as a high value-added product.

## Conclusions

In this study, crude glycerol as an abundant by-product generated in the growing biodiesel industry, was utilised for bio-based chemical production. It was adopted as the sole carbon source for SA production by an engineered *Y. lipolytica* PGC01003. This strain exhibits excellent tolerance to environmental stress in as high as 200 g L^−1^ crude glycerol to produce 43 g L^−1^ SA. We also achieved the highest fermentative SA titer of 160.2 g L^−1^ using fed-batch fermentation, comparing with other studies reported so far, with the highest SA productivity of 0.40 g L^−1^ h^−1^. Results from this study successfully demonstrated the robust SA production by *Y. lipolytica*, which is a highly efficient strategy for process integration of both biodiesel and SA production in a biorefinery unit. Further, genomic scale modification should be employed to improve its capability for fermentative SA production.

## Methods

### Strains, media and raw materials

The auxotrophic strain Po1f (Leu^−^, Ura^−^) and Po1g (Leu^−^) derived from the wild type strain W29 (ATCC 20460) were kindly provided by Professor Catherine Madzak (Institut National de la Recherche Agronomique, AgroParisTech, France) [42, 43]. Po1f was used as a recipient of strain engineering and Po1g was used as the control strain. *Escherichia coli* DH5α was used for routine subcloning and plasmid propagation. It was grown in Luria–Bertani broth (LB) containing ampicillin (50 mg L^−1^) for plasmid selection. YNBG medium containing 0.67 % (w/v) yeast nitrogen base (without amino acids and with ammonium sulfate, solarbo), 0.2 % casamino acids, and 2 % (w/v) glycerol was used for transformants screening. The yeast strains were cultivated in YPG medium which comprises 2 % pure glycerol, 1 % yeast extract and 2 % tryptone. The cultivation medium for benchtop fermentation was modified YPG medium including 50–200 g L^−1^ of crude glycerol, 1 % yeast extract, 2 % tryptone and 20 mM phosphate buffer. The carbon sources and nitrogen sources were prepared and sterilized individually. All media were sterilized at 121 °C for 20 min, and then were mixed under sterile conditions. The bioreactors were sterilized separately for 30 min at 121 °C.

Crude glycerol used in this study was provided by ASB Biodiesel (Hong Kong) Ltd. The crude glycerol contains 67.1 % glycerol, 17.2 % water and 0.13 % methanol by weight, as well as trace amount of salts precipitate.

### Gene cloning and strain construction

The strains, vectors and primers used in this work are listed in Table 3. To disrupt the TCA cycle for SA accumulation, the genes encoding succinate dehydrogenase E (*Ylsdh5*, accession number of NC_006072) in chromosome of Po1f was inserted by URA3 through homologous recombination using PUT cassette (Fig. 1). The PUT deletion cassette comprising upper homologous fragment, URA3 marker and down homologous fragment was constructed by Gibson Assembly Cloning Kit (New England Biolabs (NEB), England) based on pBluescript SK(−) with 25 base pair of homologous sequence for every adjacent gene fragment [44]. Gene fragments of shd5-up (~1000 bp) and sdh5-down (~1000 bp) were amplified from the genomic DNA of *Y. lipolytica* W29 using primer pair upper-F/upper-R and down-F/down-R, respectively. The fragments of URA3 marker was amplified from JMP113 vector using primers of ura-F/ura-R. The linearized pBluescript SK(−) vector bone was generated by PCR amplification as well using primers of v-F/v-R. All the four PCR products were mixed together along with the reaction reagents under 50 °C for enzymatic assembly for 1 h, after which the reaction reagents were all transformed into the competent cell of *E. coli* DH5α [44]. The positive transformants were picked up by colony PCR resulting plasmid pPUT. After verification by gene sequencing, the disruption cassette PUT was amplified from pPUT using primer pair upper-F/down-R and the purified PCR product was transformed into strain Po1f via LiAc method [45]. URA^+^ transformants were selected on YNBG plates. Double homologous recombinants were screened by diagnostic PCR using genomic DNA as template with two sets of primer pairs of chrom-F/ura-R and chrom-F/chrom-R. DNA sequencing was carried out to confirm the disruption of *Ylsdh5* gene. The obtained knockout mutant was designated as *Y. lipolytica* PGC01003. PrimeSTAR^®^ HS DNA polymerase (*TaKaRa*, Dalin, China) was used in all PCR reaction.

**Table 3:** Strains, plasmids and primers used in this study

Strain	Genotype or relevant features	Source
*Y. lipolytica*		
W29	MatA, Wild type	INRA
Po1g	MatA, xpr2-322, axp-2, leu2-270	INRA
Po1f	MatA, xpr2-322, axp-2, leu2-270, ura3-302	INRA
PGC01003	MatA, xpr2-322, axp-2, leu2-270, ura3-302, Δsdh5::URA3	This study
*E. coli*		
DH5α	Competent Cells	TransGen Biotech
Vectors		
pBluescript SK(−)	Ampicillin resistance	Stratagene
pPUT	pBluescript SK(−) containing the deletion cassette of PUT	This study
Primers	Sequences(5′ → 3′)	Application
v-F	AGCTCCAGCTTTTGTTCCCT	Forward primer for pBluescript vector
v-R	TCAAGCTTATCGATACCGTC	Reverse primer for pBluescript vector
Ura-F	CGCTCTAGAACTAGTGGA	Forward primer for marker
Ura-R	ACCCTCACTAAAGGGAAC	Reverse primer for marker
Upper-F	AGGTCGACGGTATCGATAAGCTTGAAGATCTTCCACTCGCTGTTC	Forward primer for up segment
Upper-R	CCTAGGATCCACTAGTTCTAGAGCGGTTCAAAGCTCGGGGTGTGT	Reverse primer for up segment
Down-F	CAGCTTTTGTTCCCTTTAGTGAGGGTGCTTACTGAGGAAGGTTCCT	Forward primer for down segment
Down-R	ACTAAAGGGAACAAAAGCTGGAGCTCAGTGTTCTGTGAACGCAAG	Reverse primer for down segment
Chrom-F	ACGACAATGGCATCGGCTCT	Forward primer for verification
Chrom-R	TCGCTTGGTCTCAGTCTCCT	Reverse primer for verification

### Shaking flask cultivation

The fermentation feature of PGC01003 was investigated in shaking flask. Seven media, including both rich media and chemical synthetic media (CM), were screened and compared for high titer SA production of PGC01003. The fermentation was carried out in 300 mL shaking flasks with 50 mL of YPG, YPD, YNBG, YNBD, CM1, CM2 and CM3 media, respectively. The components of these media were listed in Additional file 3: Table S1. YPD and YNBD contained 2 % glucose instead of pure glycerol in YPG and YNBG. Three CM media of CM1, CM2 and CM3 were based on references relating yeast organic acid fermentation [14, 46, 47].

The feasibility of *Y. lipolytica* PGC01003 strain to utilise crude glycerol as sole carbon source for fermentation was carried out in 250 mL flask with 50 mL YPG medium, and cultivated at 28 °C and 220 rpm. In the test group, the modified YPG media contained 5, 10, 20, 30, 40 and 50 g L^−1^ of crude glycerol, respectively. The same concentrations of pure glycerol were used to replace crude glycerol as control. The pH was not controlled and was dropped to around 4.0. Samples were taken periodically for measuring optical density, pH, residual glycerol and organic acids.

### Methanol inhibition experiment

To confirm whether the methanol in the crude glycerol would affect the growth and SA production of *Y. lipolytica*, the methanol inhibition experiment was performed in the 250 mL shaking flasks with 50 mL modified YPG medium contained 75 g/L crude glycerol. As the methanol in the crude glycerol was not detectable after autoclave, 0, 0.2, 2 and 20 g/L methanol (filtration sterilization) was supplemented into the autoclaved modified YPG medium, respectively. The cultures were cultivated at 28 °C and 220 rpm, and samples were taken periodically for measuring optical density, pH, residual glycerol, organic acids and methanol.

### Batch fermentation in fermenter

One colony was picked up into 5 mL 2 % YPG medium and incubated at 28 °C and 220 rpm for 24 h. Culture (1 mL) was inoculated into 50 mL 2 % YPG medium in 250 mL shaking flasks as seed culture at 28 °C and 220 rpm. Seed culture (50 mL) was inoculated into 1.0 L fermentation medium to start benchtop fermentation.

Three parameters of the fermentation, namely pH, oxygen supply and crude glycerol concentration were studied to optimize the SA production. The PGC01003 mutant was cultivated in 2.5-L Sartorius Biostat B benchtop fermenter (B. Braun Melsungen AG, Melsungen, Germany) with a modified YPG medium. All cultivations were carried out at 28 °C, and pH was regulated with 5 M NaOH. Crude glycerol was used as sole carbon source, antifoam A (Sigma, Germany) was added when necessary. Samples were taken periodically for measuring optical density, pH, residual glycerol and organic acids.

To study the effect of pH, 50 g L^−1^ crude glycerol was used as carbon source, and the agitation speed was set at 600 rpm with 2.0 L min^−1^ of aeration. The pH of the culture was controlled at 4.0, 5.0 and 6.0, respectively. The effect of oxygen supply was studied using 50 g L^−1^ glycerol as carbon source and pH at 6.0. Agitation was fixed at 600 rpm, and the aeration rate was set at 1.0, 2.0 and 3.0 L min^−1^, respectively. To study the effect of the crude glycerol concentration, experiments were carried out at pH 6.0 and 2.0 L min^−1^ of aeration with agitation fixed at 600 rpm. In these experiments, 50, 75, 100, 150 and 200 g L^−1^ crude glycerol was used as carbon source, respectively.

### Fed-batch fermentation

Fed-batch fermentation was carried out in 2.5-L benchtop fermenter with initial 1.0 L working medium, using the optimal condition with pH at 6.0, agitation rate of 600 rpm and aeration rate of 2.0 L min^−1^. The YPG medium was used as the initial batch medium with 100 g L^−1^ crude glycerol. The fermentation condition was controlled as described in “Evaluation of crude glycerol as carbon source for SA production” section. When the residual glycerol was dropped below 15 g L^−1^, 100 mL of 750 g L^−1^ crude glycerol was fed to supplement the carbon source.

### SA recovery via resin-based vacuum distillation-crystallisation

The fermentation broth was collected and centrifuged at 10,000 rpm and 4 °C for 30 min to remove the cell biomass. The trace solid residues in the supernatant were further filtrated through Whatman^®^ No.1 paper. Activated carbon (10 %, w/v) was mixed with the clean supernatant for 4 h to remove the dark brown colour of the broth. The suspension was then separated by paper filtration and a clear fermentation broth obtained was further concentrated by distillation at 55 °C for 3 h. The pH of the broth was adjusted to 2.0 using 37 % hydrochloric acid. The crystallisation of SA was carried out at 4 °C for 24 h. The final slurry was filtrated through Whatman^®^ No. 1 paper and the SA crystals were dried at 70 °C for 12 h. The residual liquid was concentrated and crystallized again. The total crystal was weighted and 2.0 g crystal was dissolved in water for purity analysis by high performance liquid chromatography (HPLC). The purity and purification yield of the recovery process are defined by Eqs. 1 and 2.

1 $$\text{Re} \text{cov} {\text{ery}}\;(\% )\; = \;\frac{\text{Total dry weight of SA in crystals}}{\text{Total weight of SA in fermentation broth}}\; \times \;100\,\%$$

2 $${\text{Purity}}\; ( {\text{\% )}}\; = \;\frac{\text{SA weight in crystals by HPLC analysis}}{\text{Total crystals weight}}\; \times \;100\,\%$$

### SDH activity assay

The *Y. lipolytica* strains were grown in 300-mL flasks, containing 50 mL of YPG with 5 % glycerol for 24 h with shaking. The cells were harvested by centrifugation, washed and suspended in 5 mL of an extracting buffer (250 mM sucrose, 1 mM EDTA, and 10 mM Tris–HCl, pH 7.2). The cells were disrupted using 3.0 g of glass beads (0.425–0.600 mm diameter; Sigma-Aldrich, St. Louis, MO) for 5 min in a 50-mL plastic tube by vortex. The mitochondrial pellet was then prepared and the SDH enzyme activity was measured as described by Yuzbashev et al. [20].

### Analytical techniques

The cell dry weight (CDW) was calculated by heating and drying of the biomass. The specific growth rate (*μ*) was calculated by:

3 $$\mu = \frac{1}{X} \times \frac{{{\text{d}}X}}{{{\text{d}}t}}$$

where *X* is CDW and *t* is fermentation time.

Residual glycerol and organic acid contents were determined by HPLC equipped with an Aminex HPX-87H column (Bio-Rad, Inc., Hercules, CA) and a refractive index detector. The analysis was performed using 5 mM H_2_SO_4_ as mobile phase at 0.6 mL min^−1^, and the column temperature was 60 °C. All samples were passed through 0.22 μm filters before loading.

## Additional files

10.1186/s13068-016-0597-8 Overview of the metabolic pathways related to succinic acid biosynthesis from glycerol in *Y. lipolytica*. The conversion of succinic acid to fumarate catalysed by succinate dehydrogenase complex (SDH) should be blocked.
10.1186/s13068-016-0597-8 Time profiles of glycerol consumption rate, SA productivity and SA yield in fed-batch fermentation. Arrows showed the feeding points.
10.1186/s13068-016-0597-8 Components of the media in shaking flasks.

## Abbreviations

SA: succinic acid
SDH: succinate dehydrogenase
TCA: tricarboxylic acid
LB: Luria–Bertani broth
CM: chemical synthetic media
CDW: cell dry weight
