# Supplementary material for: Robust succinic acid production from crude glycerol using engineered Yarrowia lipolytica
Source: Biotechnol Biofuels. 2016 Aug 30;9(1):179. doi: 10.1186/s13068-016-0597-8 (PMC5004273; doi:10.1186/s13068-016-0597-8)
Supplement: Supplementary file 1 — 10.1186/s13068-016-0597-8 Overview of the metabolic pathways related to succinic acid biosynthesis from glycerol in Y. lipolytica. The conversion of succinic acid to fumarate catalysed by succinate dehydrogenase complex (SDH) should be blocked. [file 13068_2016_597_MOESM1_ESM.doc]

### **Supplementary information**

Robust succinic acid production from crude glycerol by using engineered *Yarrowia lipolytica*

Cuijuan Gao^1, 2, 3, †^, Xiaofeng Yang^1, 4, †^, Huaimin Wang^1^, Cristina Perez Rivero^5^, Chong Li^1^, Zheyong Cui^2^, Qingsheng Qi^2^, Carol Sze Ki Lin^1^**^*^**

^1^. *School of Energy and Environment, City University of Hong Kong, Tat Chee Avenue, Kowloon, Hong Kong*

^2^. *State Key Laboratory of Microbial Technology, Shandong University, Jinan, 250100, People’s Republic of China*

^3^. *School of Life Science, Linyi University, Linyi, 276005, People’s Republic of China*

^4^. *School of Bioscience and Bioengineering, South China University of Technology, Guangzhou, 510006, People’s Republic of China*

^5^. *School of Chemical Engineering and Analytical Science, The University of Manchester, Manchester, UK*

† The authors contributed equally to this work.

* Corresponding author. School of Energy and Environment, City University of Hong Kong, Tat Chee Avenue, Kowloon, Hong Kong. Tel.: +852 3442 7497; Fax: +852 3442 0688. E-mail address: carollin@cityu.edu.hk (C.S.K. Lin).


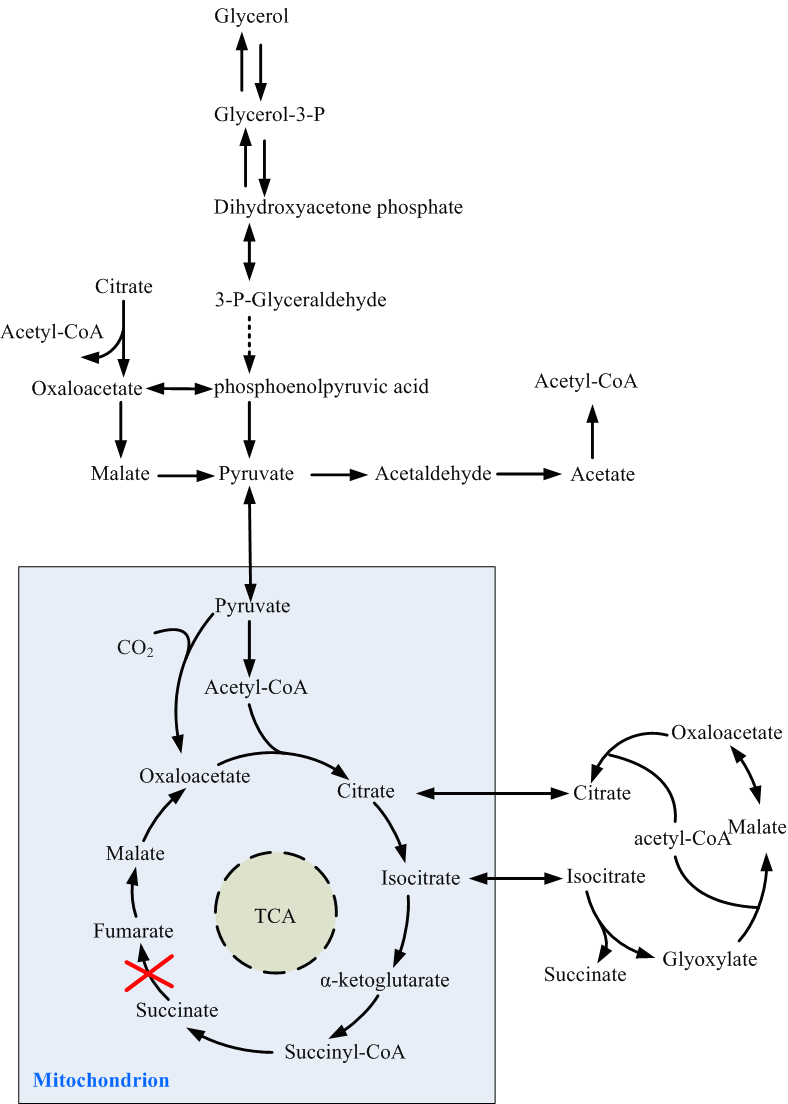


**Figure S1.** Overview of the metabolic pathways related to succinic acid biosynthesis from glycerol in *Y. lipolytica*. The conversion of succinic acid to fumarate catalysed by succinate dehydrogenase complex (SDH) should be blocked.
