# Supplementary material for: Robust succinic acid production from crude glycerol using engineered Yarrowia lipolytica
Source: Biotechnol Biofuels. 2016 Aug 30;9(1):179. doi: 10.1186/s13068-016-0597-8 (PMC5004273; doi:10.1186/s13068-016-0597-8)
Supplement: Supplementary file 2 — 10.1186/s13068-016-0597-8 Time profiles of glycerol consumption rate, SA productivity and SA yield in fed-batch fermentation. Arrows showed the feeding points. [file 13068_2016_597_MOESM2_ESM.doc]

### **Supplementary information**

Robust succinic acid production from crude glycerol by using engineered *Yarrowia lipolytica*

Cuijuan Gao^1, 2, 3, †^, Xiaofeng Yang^1, 4, †^, Huaimin Wang^1^, Cristina Perez Rivero^5^, Chong Li^1^, Zheyong Cui^2^, Qingsheng Qi^2^, Carol Sze Ki Lin^1^**^*^**

^1^. *School of Energy and Environment, City University of Hong Kong, Tat Chee Avenue, Kowloon, Hong Kong*

^2^. *State Key Laboratory of Microbial Technology, Shandong University, Jinan, 250100, People’s Republic of China*

^3^. *School of Life Science, Linyi University, Linyi, 276005, People’s Republic of China*

^4^. *School of Bioscience and Bioengineering, South China University of Technology, Guangzhou, 510006, People’s Republic of China*

^5^. *School of Chemical Engineering and Analytical Science, The University of Manchester, Manchester, UK*

† The authors contributed equally to this work.

* Corresponding author. School of Energy and Environment, City University of Hong Kong, Tat Chee Avenue, Kowloon, Hong Kong. Tel.: +852 3442 7497; Fax: +852 3442 0688. E-mail address: carollin@cityu.edu.hk (C.S.K. Lin).


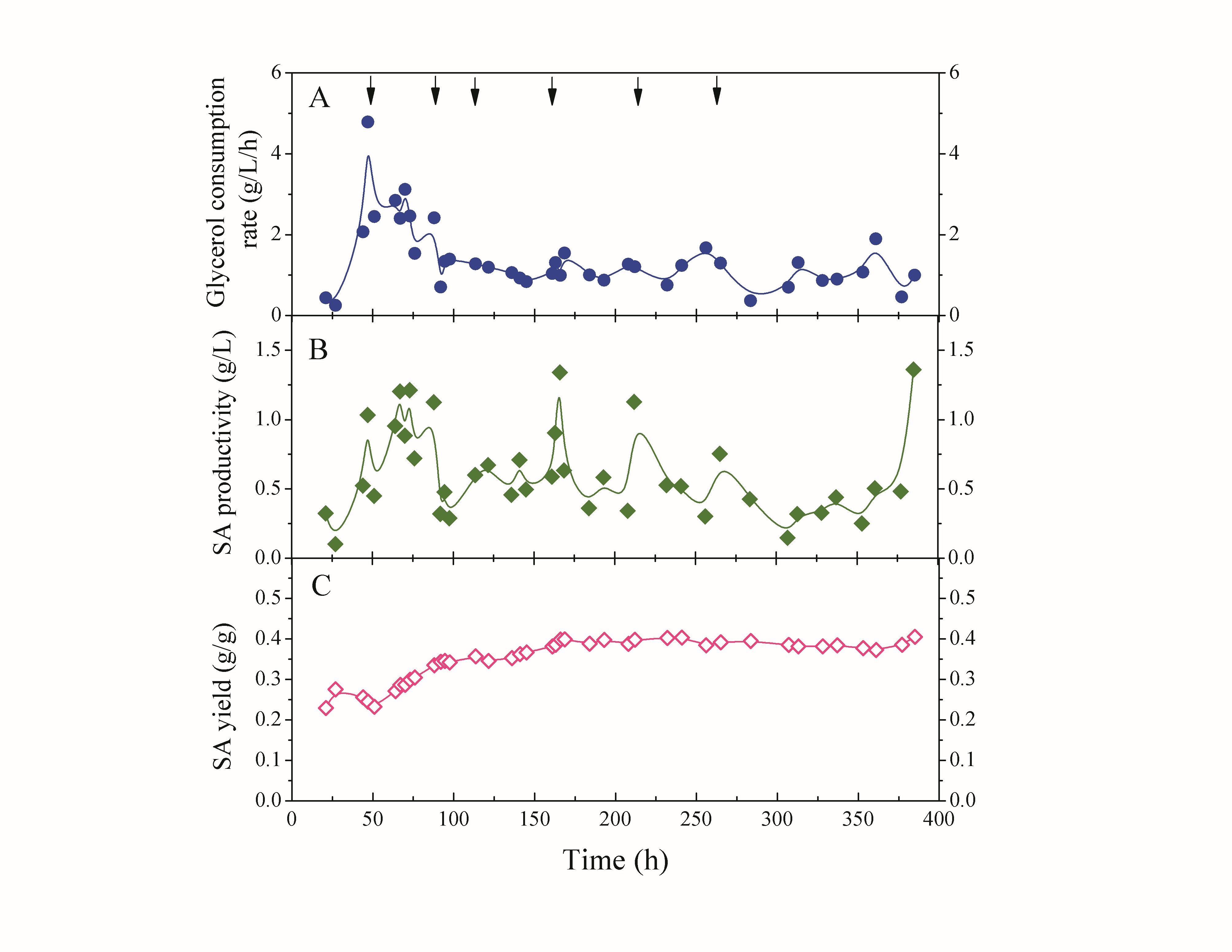


**Figure S2.** Time profiles of glycerol consumption rate, SA productivity and SA yield in fed-batch fermentation. Arrows showed the feeding points.
