# Supplementary material for: Robust succinic acid production from crude glycerol using engineered Yarrowia lipolytica
Source: Biotechnol Biofuels. 2016 Aug 30;9(1):179. doi: 10.1186/s13068-016-0597-8 (PMC5004273; doi:10.1186/s13068-016-0597-8)
Supplement: Supplementary file 3 — 10.1186/s13068-016-0597-8 Components of the media in shaking flasks. [file 13068_2016_597_MOESM3_ESM.doc]

### **Supplementary information**

Robust succinic acid production from crude glycerol by using engineered *Yarrowia lipolytica*

Cuijuan Gao^1, 2, 3, †^, Xiaofeng Yang^1, 4, †^, Huaimin Wang^1^, Cristina Perez Rivero^5^, Chong Li^1^, Zheyong Cui^2^, Qingsheng Qi^2^, Carol Sze Ki Lin^1^**^*^**

^1^. *School of Energy and Environment, City University of Hong Kong, Tat Chee Avenue, Kowloon, Hong Kong*

^2^. *State Key Laboratory of Microbial Technology, Shandong University, Jinan, 250100, People’s Republic of China*

^3^. *School of Life Science, Linyi University, Linyi, 276005, People’s Republic of China*

^4^. *School of Bioscience and Bioengineering, South China University of Technology, Guangzhou, 510006, People’s Republic of China*

^5^. *School of Chemical Engineering and Analytical Science, The University of Manchester, Manchester, UK*

† The authors contributed equally to this work.

* Corresponding author. School of Energy and Environment, City University of Hong Kong, Tat Chee Avenue, Kowloon, Hong Kong. Tel.: +852 3442 7497; Fax: +852 3442 0688. E-mail address: carollin@cityu.edu.hk (C.S.K. Lin).

**Table S1** Components of the media in shaking flasks.

| **Media** | **Components** |
| --- | --- |
| **YPD** | 10 g/L Yeast extract, 20 g/L Tryptone, 20 g/L Glucose |
| **YNBD** | 6.7 g/L Yeast nitrogen based (w/o amino acid), 2 g/L Casamino acid, 20g/L Glucose |
| **YPG** | 10 g/L Yeast extract, 20 g/L Tryptone, 20 g/L Glycerol |
| **YNBG** | 6.7 g/L Yeast nitrogen based (w/o amino acid), 2 g/L Casamino acid, 20 g/L Glycerol |
| **CM1** | 6.7 g/L Yeast nitrogen based (w/o amino acid), 5.0 g/L Yeast extract, 6.0 g/L KH_2_PO_4_, 2.0 g/L K_2_HPO_4_, 1.5 g/L MgSO_4_.7H_2_O, 1.0 mg/L Thiamine hydrochloride and 20 g/L Glycerol |
| **CM2** | 5.0 g/L (NH_4_)_2_ SO_4_, 3.0 g/L KH_2_PO_4_, 0.5 g/L MgSO_4_.7H_2_O, 0.45 g/L CaCl_2_.6H_2_O, 1.0 mg/L Thiamine hydrochloride, 1 ml Trace element, 20g/L Glycerol |
| **CM3** | 3.0 g/L (NH_4_)_2_ SO_4_, 3.0 g/L KH_2_PO_4_, 1.2 g/L MgSO_4_, 0.5 g/L NaCl, 0.1 g/L K_2_HPO_4_, 1.0 mg/L Thiamine hydrochloride, 20g/L Glycerol |
